# Overactivated neddylation pathway in human hepatocellular carcinoma

**DOI:** 10.1002/cam4.1578

**Published:** 2018-05-30

**Authors:** Jian Yu, Wei‐long Huang, Qing‐guo Xu, Ling Zhang, Shu‐han Sun, Wei‐ping Zhou, Fu Yang

**Affiliations:** ^1^ The Third Department of Hepatic Surgery Eastern Hepatobiliary Surgery Hospital Second Military Medical University Shanghai China; ^2^ Key Laboratory of Signaling Regulation and Targeting Therapy of Liver Cancer (SMMU) Ministry of Education Shanghai China; ^3^ Shanghai Key Laboratory of Hepatobiliary Tumor Biology (EHBH) Shanghai China; ^4^ Department of Medical Genetics Second Military Medical University Shanghai China

**Keywords:** NAE1, NEDD8, UBE2M, UCHL1

## Abstract

Dysregulation of the neddylation pathway is related to various cancers. However, the specific role of the neddylation pathway in human hepatocellular carcinoma (HCC) remains largely unclear. In this study, the neddylation pathway in HCC and adjacent noncancerous liver (ANL) tissues was evaluated by immunohistochemistry (IHC), Western blotting, and qRT‐PCR (quantitative real‐time polymerase chain reaction). The results showed that the entire neddylation pathway, including NEDD8 (the IHC staining of NEDD8 represents the global‐protein neddylation), E1 NEDD8‐activating enzymes (NAE1 and UBA3), E2 NEDD8‐conjugating enzymes (UBE2F and UBE2M), E3 NEDD8‐ligases (MDM2, RBX1 and RNF7), and deneddylation enzymes (COPS5, UCHL1 and USP21), was overactivated in HCC. Furthermore, the upregulation of NEDD8 in HCC was correlated with aggressive characteristics and was an independent risk factor for overall survival (OS) and recurrence‐free survival (RFS) in patients with HCC after hepatectomy. The upregulation of NAE1, UBE2M, and UCHL1 in HCC was associated with aggressive characteristics and poor OS and RFS in patients with HCC after hepatectomy. In conclusion, our research reveals that the entire neddylation pathway is overactivated in HCC and associated with clinical characteristics and prognosis of patients with HCC.

## 1 INTRODUCTION

Approximately 600 000 people die of hepatocellular carcinoma (HCC) every year, which makes HCC one of the most common causes of cancer‐related deaths worldwide.^1^ However, the mechanism of the development and progression of HCC is not clear.^2^ The identification of the mechanism involved in the process of HCC would identify not only novel therapeutic targets but also prognostic and diagnostic markers.^2^, ^3^ Protein neddylation is a newly characterized post‐translational modification of a ubiquitin‐like protein named neural precursor cell‐expressed developmentally downregulated protein 8 (NEDD8).^4^, ^5^, ^6^ Like the ubiquitin pathway, the neddylation pathway involves NEDD8‐specific E1, E2, E3, and deneddylation enzymes.^7^, ^8^ NEDD8 is first synthesized as a precursor without any known biological function; then, the NEDD8 precursor is hydrolyzed by the deneddylation enzymes at the conserved C‐terminal Gly76 residue.^6^, ^9^, ^10^ Mature NEDD8 is then covalently linked to target proteins via the carboxy‐terminal glycine residue in a series of reactions catalyzed by E1‐, E2‐, and E3‐like enzymes.^11^, ^12^, ^13^ NEDD8 modification regulates the target protein conformation, the subcellular localization, and the stability and binding affinity to DNA and proteins.^14^, ^15^ Moreover, the dysregulation of the neddylation pathway is related to tumors,^8^ neurodegenerative disorders,^16^ inflammation,^17^, ^18^ immunodeficiency,^19^ and heart failure^20^. Recently, the oncoprotein Hu antigen R (HuR) was shown to be neddylated in HCC,^21^ which further highlights a pivotal role for neddylation in HCC carcinogenesis and progression. What’s more, LKB1 and Akt, stabilized by neddylation, regulates energy metabolism in human HCC.^22^ Additionally, using the Nedd8‐activating enzyme inhibitor MLN4924, the inhibition of neddylation pathway inhibits the growth of HCC cells by inducing autophagy and apoptosis.^23^ However, the expression of NEDD8, neddylation, and deneddylations enzymes in human HCC and its clinical meaning have not been studied systematically before.

Here, we detected the expression of the entire neddylation pathway in human HCC and explored its association with clinical features and prognosis of patients with HCC after hepatectomy.

## 2 MATERIALS AND METHODS

### 2.1 Patients and specimens

In total, 509 pairs of HCC and corresponding adjacent noncancerous liver (ANL) tissues were obtained from surgical resections of patients without preoperative treatment at Eastern Hepatobiliary Surgery Hospital (Shanghai, China). The inclusion criteria have been reported previously,^24^ that is, meet the clinical diagnosis criteria of HCC from the AASLD Practice Guidelines (http://www.aasld.org/practiceguidelines/Documents/Bookmarked%20Practice%20Guidelines/HCCUpdate2010.pdf); preoperative World Health Organization performance status of 0‐1; Child‐Pugh class A; no distant metastasis, visualizable ascites, or encephalopathy; no chemotherapy or radiotherapy before surgery; curative resection (R0 resection); and resected lesions identified as HCC on pathological examination. Human specimen collection was approved by the Ethics Committee of Eastern Hepatobiliary Surgery Hospital. Written informed consent was obtained from each patient according to the policies of the committee. The resected samples were identified by 2 pathologists independently. The samples used in PCR and Western blotting analysis were fresh frozen, and the samples used in IHC (immunohistochemistry) were FFPE (formalin fixed and paraffin embedded). Among them, 306 pairs (cohort 1) were used for the detection of NEDD8 and the analysis of the correlation between NEDD8 expression and outcome of patients with HCC after hepatectomy, 40 pairs (cohort 2) were used for the detection of NEDD8 and all the genes in the neddylation pathway, and the other 163 pairs (cohort 3) were used for quantification of NAE1, UBE2M, and UCHL1 and analysis of the correlation between their expression and outcome of patients with HCC after hepatectomy. The detailed clinicopathological features are described in Table S1. The 7th edition of the AJCC/UICC TNM staging system for HCC was used in this study. The samples of cohorts 1, 2, and 3 were obtained in 2005 to 2010, 2016, and 2010 to 2011, respectively.

### 2.2 Follow‐up

The follow‐up examinations were performed as the previous study.^25^ The patients of cohort 1 and cohort 3 received check‐ups every 2‐3 months after surgery during the first 24 months and every 3‐6 months thereafter until January 2013 and November 2016, respectively. The median follow‐up period of cohort 1 and cohort 3 was 55.0 and 45.2 months, respectively. Physicians who were blinded to the study performed the follow‐up examinations. Serum AFP levels and abdominal ultrasound examinations were performed every month during the first year after surgery and every 3‐6 months thereafter. Computed tomography and/or magnetic resonance imaging were performed every 3‐6 months or when a recurrence was suspected. The diagnosis of recurrence was based on the diagnosis criteria from the AASLD Practice Guidelines (http://www.aasld.org/practiceguidelines/Documents/Bookmarked%20Practice%20Guidelines/HCCUpdate2010.pdf). Once recurrence was confirmed, further treatment was implemented based on the tumor diameter, the number of tumors, the location of the tumor, and the extent of vessel invasion as well as liver function and performance statuses. Recurrence‐free survival (RFS) was calculated from the date of tumor resection until the detection of tumor recurrence, death from a cause other than HCC, or the last follow‐up visit. The overall survival (OS) was defined as the length of time between surgery and either the death of the patient or the last follow‐up visit.

### 2.3 RNA extraction and quantitative real‐time PCR (qRT‐PCR)

Total RNA was isolated using the TRIzol^®^ LS Reagent (10296‐010, Thermo Fisher Scientific). To generate the cDNA, the M‐MLV Reverse Transcriptase (28025‐013, Thermo Fisher Scientific) and random primers were used according to the manufacturer’s guidelines. To perform the quantitative real‐time PCR, we used the StepOne^™^ Real‐Time PCR System (Applied Biosystems, Foster City, USA) with SYBR^®^ Green (Takara, Dalian, China). β‐actin was used as the endogenous control. The gene‐specific primers are shown in Table S2.

### 2.4 Western blotting analysis

Total protein was extracted from snap‐frozen tissues with RIPA Lysis Buffer and PMSF (Beyotime Co., China) according to the manufacturer’s instructions. Antibody binding was detected with an Odyssey infrared scanner (Li‐Cor Biosciences Inc.) as previously described.^26^ β‐actin was used as a loading control. The representative results of the same trend in 3 independent experiments are presented. The antibodies are listed in Table S3.

### 2.5 Immunohistochemical staining of the HCC tissue microarray

The tissue microarrays and IHC were performed as previously described.^25^ After heating, the sections were incubated with primary antibody for 30 min and then with the secondary antibody for an hour at room temperature. These sections were finally counterstained with hematoxylin and observed under a microscope after developing in the diaminobenzidine solution. The staining was evaluated by 3 specialized pathologists and was performed without any knowledge of the patient characteristics. For H‐score^27^, ^28^ the assessment fields were at ×200 magnification, and the staining intensity in the malignant cells was scored as 0, 1, 2, or 3 corresponding to the presence of negative, weak, intermediate, and strong brown staining, respectively. The total number of cells in each field and the number of cells stained at each intensity were counted. The average percentage of positive cells was calculated, and the following formula was applied: H‐score = (% of cells stained at intensity category 1 × 1) + (% of cells stained at intensity category 2 × 2) + (% of cells stained at intensity category 3 × 3). H‐scores between 0 and 300 were obtained, where 0 was equal to 100% of cells stained negatively, and 300 was equal to 100% of cells stained strongly. The median of the H‐scores was used as the cutoff distinguishing low expression from high expression.

### 2.6 Statistical analysis

All statistical analyses were performed using SPSS version 19.0 software (SPSS, Inc., Chicago, IL). For comparisons, the chi‐squared test and Wilcoxon signed‐rank test were performed, as appropriate. Correlations were measured by Spearman’s correlation analysis. The survival curves were calculated using the Kaplan‐Meier method, and the differences were assessed by a log‐rank test. Univariate and multivariate analyses were used according to the Cox regression model. *P *<* *.05 was considered statistically significant.

## 3 RESULTS

### 3.1 Activation status of NEDD8 in HCC

**Figure 1:**
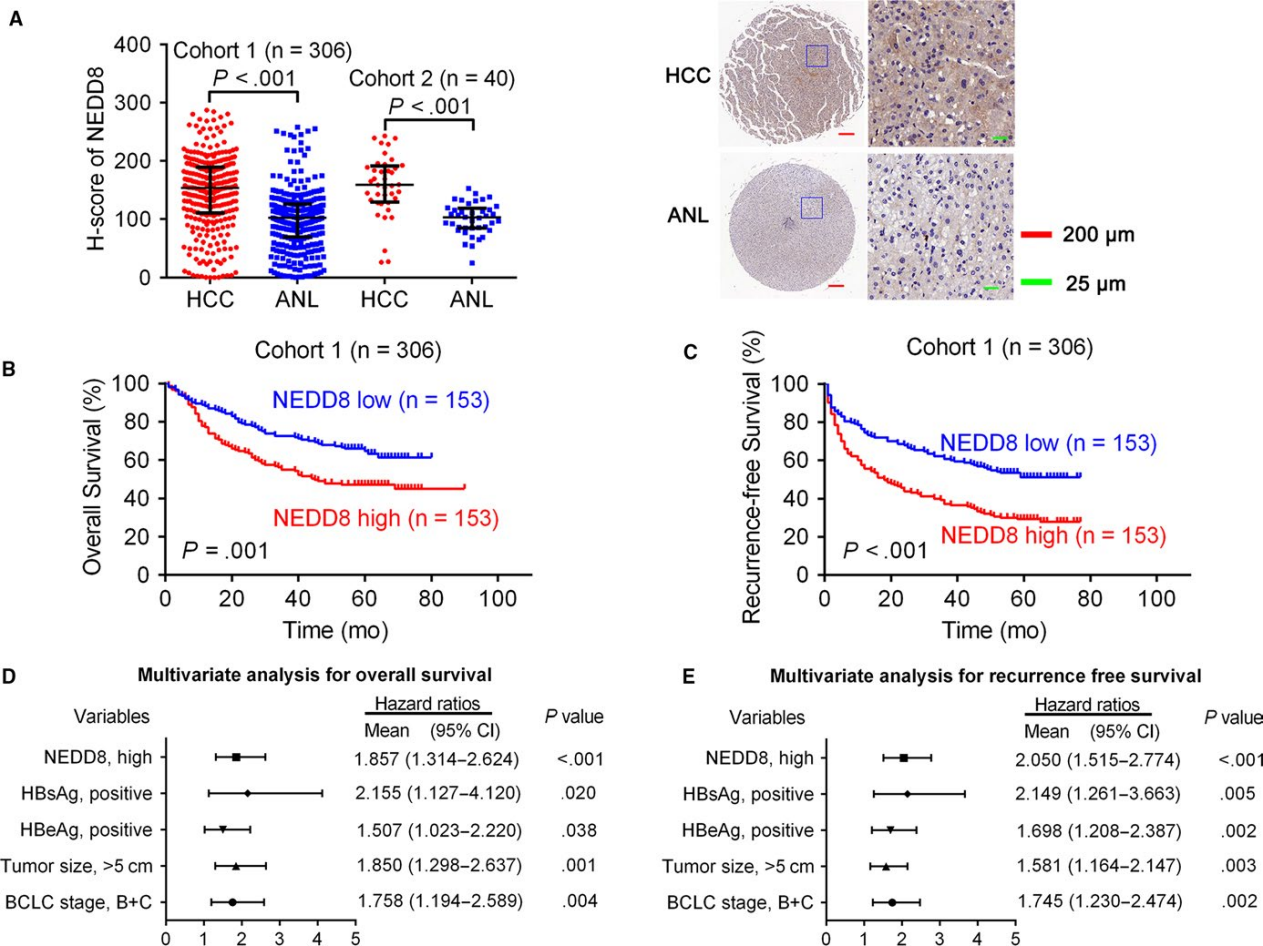
Activation status of NEDD8 in HCC. A, IHC stains of NEDD8. (Left) H‐score of NEDD8 in 346 pairs of HCC and ANL tissues. (Right) Representative samples. Horizontal lines represent the medians, and whiskers represent the 25th and 75th percentiles. The Wilcoxon signed‐rank test was used. B, C, The Kaplan‐Meier analysis of the overall survival and disease‐free survival of patients with HCC according to the expression of NEDD8 in HCC tissues. The log‐rank test was used. D, E, Multivariate analysis of hazard ratios for overall survival and disease‐free survival is shown

The expression of NEDD8 represents global‐protein neddylation. By IHC using a NEDD8 specific antibody, we detected the expression of NEDD8 in 346 pairs of HCC and ANL tissues (Cohort 1: 306 pairs, Cohort 2:40 pairs). It showed that the expression of NEDD8 was significantly upregulated in HCC tissues (Figure 1A). Previous studies have shown that HuR was stabilized by neddylation^21^ and overexpressed^21^, ^27^ in human HCC. LKB1 and Akt were also stabilized by neddylation in human HCC.^22^ However, the neddylation of Cullin 1 (the first and best characterized NEDD8 substrate^8^, ^28^) in human HCC tissues has not been reported before. Therefore, using Western blotting, we detected the expression of Cullin 1 in 10 pairs of HCC and ANL tissues. It showed that the neddylation level of Cullin 1 in HCC was higher than that in ANL tissues (Figure S1). We next sought to determine whether NEDD8 expression in HCC was associated with specific clinicopathological characteristics. The median expression level was used as the cutoff, which divided the 346 patients with HCC in Cohort 1 and Cohort 2 into a low‐NEDD8‐expression group (n = 173) and a high‐NEDD8‐expression group (n = 173). We found that a higher NEDD8 expression level was significantly correlated with positive HBeAg (hepatitis B e antigen), poorer Edmondson’s grade, and the presence of microvascular invasion (Table 1). Notably, HBeAg‐low expression was associated with NEDD8‐high expression. However, it did not mean the causal relationship between them; this awaits further exploration whether they can influence each other. Furthermore, Kaplan‐Meier’s survival curves showed that the HCC patients with a higher NEDD8 expression in HCC had poorer overall survival (OS, *P *=* *.001) and recurrence‐free survival (RFS, *P *<* *.001) after hepatectomy (Figure 1B,C).

**Table 1:** Clinical characteristics of 346 patients with HCC (Cohort 1 and 2) according to NEDD8 expression level

Variables	NEDD8		*P*‐value
	Low	High	
All cases	173	173	
Age, y, ≥60:<60	34:139	41:132	.361
Gender, male/female	132:18	137:19	.962
HBsAg, positive/negative	152:21	147:26	.433
HBeAg, positive/negative	27:146	47:126	.009*
AFP, μg/L, >20:≤20	108:65	111:62	.738
Liver cirrhosis, with/without	125:48	132:41	.389
Tumor number, multiple:solitary	34:139	30:143	.580
Tumor size, cm, >5:≤5	87:86	88:85	.914
Edmondson’s grade, III + IV:I + II	166:7	146:27	<.001*
Pathological satellite, present/absent	129:44	116:57	.124
Microvascular invasion, present:absent	135:38	61:112	<.001*
TNM stage, II + III:I	45:128	34:139	.159
BCLC stage, B + C:0 + A	34:117	29:127	.394

AFP, alpha‐fetoprotein; BCLC, Barcelona Clinic Liver Cancer; HBsAg, hepatitis B surface antigen; HBeAg, hepatitis B e antigen; HCC, hepatocellular carcinoma; TNM, tumor‐node‐metastasis.

*P <* *.05 by the chi‐squared test.

Univariate analyses revealed that NEDD8 expression, HBsAg (hepatitis B surface antigen), HBeAg, tumor number, tumor size, microvascular invasion, TNM, and BCLC stages were significantly correlated with OS, and NEDD8 expression, HBsAg, HBeAg, tumor number, tumor size, Edmondson’s grade, microvascular invasion, TNM, and BCLC stages were significantly correlated with RFS (Table S4). Using multicollinearity analyses, we found that there existed high multicollinearity (variance inflation >10) between tumor number and TNM stage (Tables S5 and S6). As the TNM staging of HCC was based on tumor size, tumor number, and vascular invasion, we excluded TNM stage. We then did multicollinearity analyses again and found no significant multicollinearity (all variance inflations were less than 5) (Tables S7 and S8). After excluding TNM stage, these clinicopathological characteristics were further applied for multiple analyses for OS and RFS, respectively. The multivariate analyses indicated that higher NEDD8 expression, together with positive HBsAg, positive HBeAg, larger tumor size, and poorer BCLC stage, was an independent risk factor for both OS and RFS (Figure 1D,E, Table S9).

### 3.2 Activation status of neddylation enzymes in HCC

**Figure 2:**
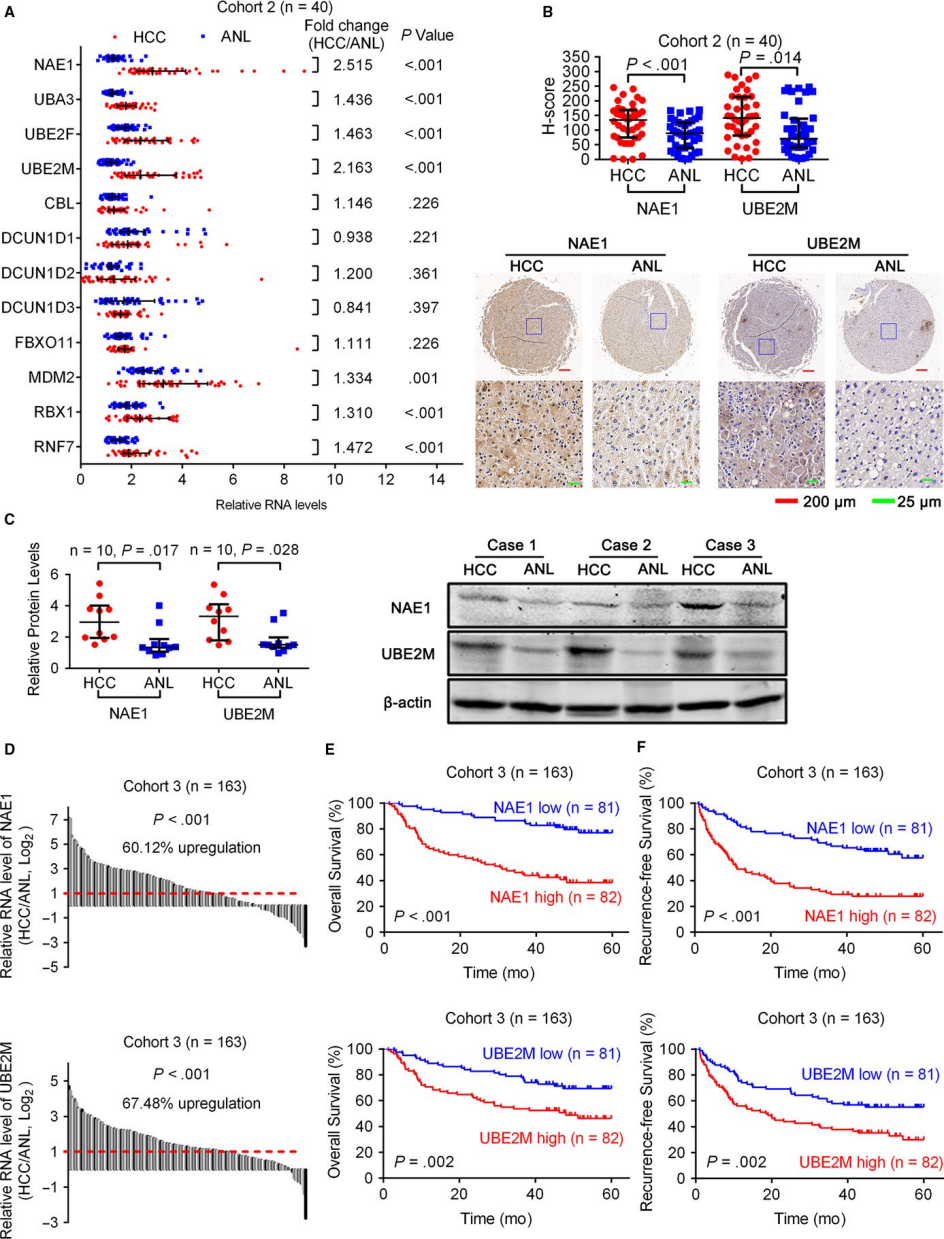
Expression of neddylation enzymes in HCC. A, qRT‐PCR showed the mRNA levels of neddylation enzymes in 40 pairs of HCC and ANL tissues. β‐actin was used as the endogenous control. B, IHC stains of NAE1 and UBE2M. (Top) H‐score of NAE1 and UBE2M in 40 pairs of HCC and ANL tissues. (Bottom) Representative samples. C, (Left) Western blotting analysis showed the protein expression of NAE1 and UBE2M in 10 pairs of HCC and ANL tissues. (Right) Representative samples. D, qRT‐PCR showed the mRNA levels of NAE1 and UBE2M in 163 pairs of HCC and ANL tissues. β‐actin was used as the endogenous control. E, F, The Kaplan‐Meier analysis of the overall survival and disease‐free survival of patients with HCC according to the expression of NAE1 or UBE2M in HCC tissues. The log‐rank test was used. For (A‐C), horizontal lines represent the medians, and whiskers represent the 25th and 75th percentiles. For (A‐D), the Wilcoxon signed‐rank test was used

Neddylation enzymes consist of E1 NEDD8‐activating enzymes (NAE1 and UBA3), E2 NEDD8‐conjugating enzymes (UBE2F and UBE2M), and E3 NEDD8‐ligases (CBL, DCUN1D1, DCUN1D2, DCUN1D3, FBXO11, MDM2, RBX1, and RNF7) in humans.^8^ Notably, UBE2M, DCUN1D1, DCUN1D2, DCUN1D3, and RNF7 are also known as UBC12, DCNL1, DCNL2, DCNL3, and RBX2, respectively. Using qRT‐PCR, we detected the expression of these neddylation enzymes in 40 pairs of HCC and ANL tissues. The ∆*C*_*t*_ (compared to β‐actin) values of these twelve neddylation enzymes were shown in Figure S2. It showed that NAE1, UBA3, UBE2F, UBE2M, MDM2, RBX1, and RNF7 were significantly upregulated in HCC tissues (Figure 2A). Among them, MDM2 had been reported to be upregulated in HCC,^29^ which was consistent with our results. As NAE1 and UBE2M were the most severely upregulated (more than twofold changes), we further detected the protein expression of NAE1 and UBE2M in paired HCC and ANL tissues. IHC and Western blotting analyses showed that the protein expression of NAE1 and UBE2M was also upregulated in HCC (Figure 2B,C).

In another cohort (Cohort 3, 163 HCC patients), qRT‐PCR confirmed that NAE1 and UBE2M were significantly upregulated in HCC (Figure 2D). Higher NAE1 expression was correlated with larger tumor size, poorer Edmondson’s grade, presence of microvascular invasion, poorer TNM, and poorer BCLC stage, and higher UBE2M expression was correlated with multiple tumors and the presence of microvascular invasion (Table 2). Moreover, Kaplan‐Meier’s survival curves showed that the HCC patients with higher NAE1 or UBE2M expression in HCC had poorer OS and RFS after hepatectomy (Figure 2E,F).

**Table 2:** Clinical characteristics of 203 patients with HCC (Cohorts 2 and 3) according to NAE1, UBE2M, or UCHL1 expression level

Variables	NAE1			UBE2M			UCHL1		
	Low	High	*P*‐value	Low	High	*P*‐value	Low	High	*P*‐value
All cases	101	102		101	102		101	102	
Age, years, ≥60:<60	20:81	25:77	.419	26:75	19:83	.222	19:82	26:76	.252
Gender, male/female	84:17	87:15	.678	87:14	84:18	.459	86:15	85:17	.723
HBsAg, positive/negative	81:20	80:22	.756	77:24	84:18	.282	84:17	77:25	.177
HBeAg, positive/negative	28:73	24:78	.494	26:75	26:76	.967	26:75	26:76	.967
AFP, μg/L, >20:≤20	61:40	73:29	.093	61:40	73:29	.093	69:32	65:37	.490
Liver cirrhosis, with/without	67:34	56:46	.096	64:37	59:43	.421	70:31	53:49	**.011** a
Tumor number, multiple:solitary	17:84	16:86	.825	10:91	23:79	.015^a^	19:82	14:88	.326
Tumor size, cm, >5:≤5	46:55	67:35	.004^a^	55:46	58:44	.730	52:49	61:41	.233
Edmondson’s grade, III + IV:I + II	77:24	91:11	.014^a^	80:21	88:14	.183	81:20	87:15	.337
Pathological satellite, present/absent	39:62	45:57	.426	42:59	42:60	.953	39:62	45:57	.426
Microvascular invasion, present:absent	26:75	42:60	.020^a^	27:74	41:61	.042^a^	27:74	41:61	**.042** a
TNM stage, II + III:I	49:52	65:37	.029^a^	56:45	58:44	.839	56:45	58:44	.839
BCLC stage, B + C:0 + A	51:50	66:36	.040^a^	56:45	61:41	.530	64:37	53:49	.100

AFP, alpha‐fetoprotein; BCLC, Barcelona Clinic Liver Cancer; HBsAg, hepatitis B surface antigen; HBeAg, hepatitis B e antigen; HCC, hepatocellular carcinoma; TNM, tumor‐node‐metastasis.

a *P *<* *.05 by the chi‐squared test.

**Figure 3:**
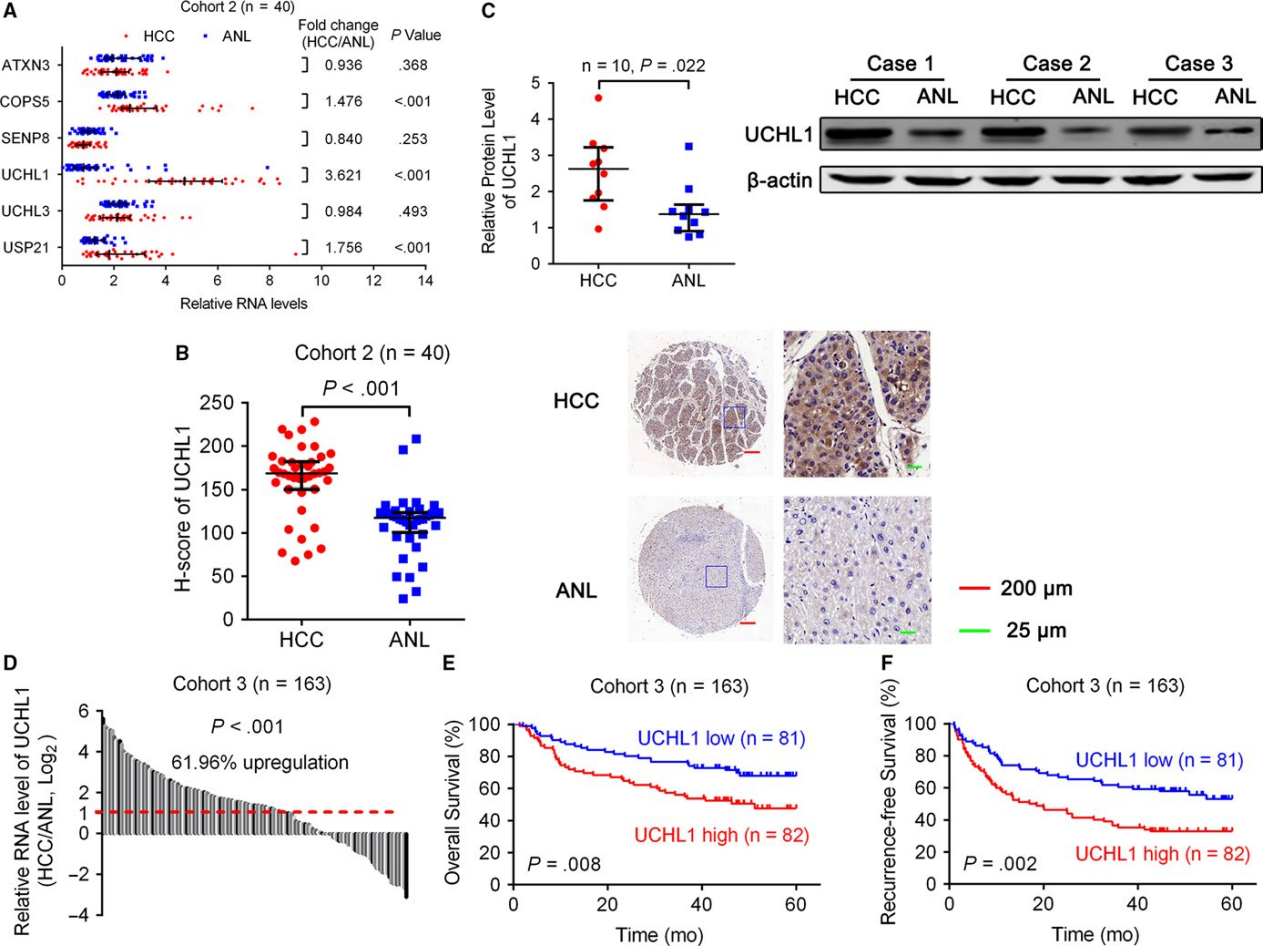
Expression of deneddylation enzymes in HCC. A, qRT‐PCR showed the mRNA levels of deneddylation enzymes in 40 pairs of HCC and ANL tissues. β‐actin was used as the endogenous control. B, IHC stains of UCHL1. (Left) H‐score of UCHL1 in 40 pairs of HCC and ANL tissues. (Right) Representative samples. C, (Left) Western blotting analysis showed the protein expression of UCHL1 in 10 pairs of HCC and ANL tissues. (Right) Representative samples. D, qRT‐PCR showed the mRNA levels of UCHL1 in 163 pairs of HCC and ANL tissues. β‐actin was used as the endogenous control. E, F, The Kaplan‐Meier analysis of the overall survival and disease‐free survival of patients with HCC according to the expression of UCHL1 in HCC tissues. The log‐rank test was used. For (A‐C), horizontal lines represent the medians, and whiskers represent the 25th and 75th percentiles. For (A‐D), the Wilcoxon signed‐rank test was used

Furthermore, we analyzed the expression of these genes in 214 paired HBV‐related human HCC and ANL tissues from a Chinese cohort^30^ from Gene Expression Omnibus (https://www.ncbi.nlm.nih.gov/geo/) (GSE14520, GPL3921) using GEO2R^31^. We found that NAE1 (adjusted *P*‐value = 2.18E−41, fold change = 1.760), UBA3 (adjusted *P*‐value = 4.47E−22, fold change = 1.410), UBE2M (adjusted *P*‐value = 3.45E−24, fold change = 1.561), RBX1 (adjusted *P*‐value = 3.38E−53, fold change = 1.651), and RNF7 (adjusted *P*‐value = 7.64E−30, fold change = 1.481) were also upregulated in HCC in this microarray data (Table S10). In addition, higher NAE1 or UBE2M expression in liver cancer indicated poorer survival (Figure 3A,B) according to an interactive open‐access database The Human Protein Atlas^32^ (https://www.proteinatlas.org/pathology). All these findings further confirmed our results.

**Figure 4:**
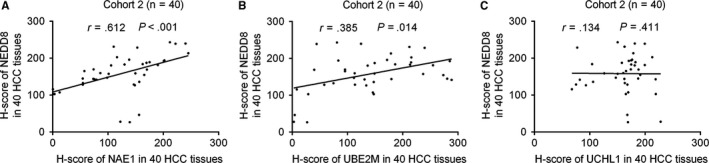
The correlations between the expression of NEDD8 and NAE1, UBE2M, or UCHL1 in HCC tissues. The correlations between the expression of NEDD8 and NAE1 (A), UBE2M (B), or UCHL1 (C) in 40 HCC tissues were measured by Spearman correlation analysis

### 3.3 Activation of the deneddylation system in HCC

Deneddylation enzymes consist of ATXN3, COPS5 (also known as CSN5 or JAB1), SENP8 (also known as NEDP1 or DEN1), UCHL1, UCHL3, and USP21 in humans.^8^ Using qRT‐PCR, we detected the expression of these deneddylation enzymes in 40 pairs of HCC and ANL tissues. The △*C*_*t*_ (compared to β‐actin) values of these 6 deneddylation enzymes in HCC and ANL tissues were shown in Figure S2. It showed that COPS5, UCHL1, and USP21 were significantly upregulated in HCC tissues (Figure 3A). Among them, COPS5 had been reported to be upregulated in HCC,^33^ which was consistent with our results. As UCHL1 was the most severely upregulated (more than twofold changes), we further detected the protein expression of UCHL1 in paired HCC and ANL tissues. IHC and Western blotting analyses showed that the protein expression of UCHL1 was also upregulated in HCC (Figure 3B,C).

In another cohort (Cohort 3, 163 HCC patients), qRT‐PCR confirmed that UCHL1 was significantly upregulated in HCC (Figure 3D). Higher UCHL1 expression was correlated with liver cirrhosis and presence of microvascular invasion (Table 2). Moreover, Kaplan‐Meier’s survival curves showed that the HCC patients with higher UCHL1 expression in HCC had poorer OS and RFS after hepatectomy (Figure 3E,F).

Furthermore, we analyzed the expression of these genes in 214 paired HBV‐related human HCC and ANL tissues from a Chinese cohort^30^ from Gene Expression Omnibus (https://www.ncbi.nlm.nih.gov/geo/) (GSE14520, GPL3921) using GEO2R^31^. We found that COPS5 (adjusted *P*‐value = 2.37E−40, fold change = 1.806), UCHL1 (adjusted *P*‐value = 3.15E−07, fold change = 1.509), and USP21 (adjusted *P*‐value = 1.25E−29, fold change = 1.511) were also upregulated in HCC in this microarray data (Table S10). In addition, higher UCHL1 expression in liver cancer indicated poorer survival (Figure S1C) according to an interactive open‐access database The Human Protein Atlas^32^ (https://www.proteinatlas.org/pathology). All these findings further confirmed our results.

### 3.4 The correlations between the expression of NEDD8 and NAE1, UBE2M, or UCHL1 in HCC tissues

We analyzed the correlations between the H‐scores of NEDD8 and NAE1, UBE2M, or UCHL1 in 40 HCC tissues from Cohort 2. The results showed that the expression of NEDD8, which represents global‐protein neddylation, was positively correlated with NAE1 and UBE2M, but not UCHL1.

## 4 DISCUSSION

It had been reported that MLN4924, a NAE1 inhibitor, induced autophagy and apoptosis to suppress liver cancer cell growth.^23^ In this study, we found that NEDD8 (represents global‐protein neddylation) and NAE1 were upregulated in HCC, that the upregulation of NEDD8 and NAE1 were associated with aggressive clinicopathological characteristics and poorer prognosis, and that the expression of NEDD8 and NAE1 in HCC was positively correlated.

Moreover, we found that other neddylation enzymes (UBA3, UBE2F, UBE2M, MDM2, RBX1, and RNF7) were also upregulated in HCC, and that the expression of NEDD8 and UBE2M in HCC was also positively correlated. Therefore, inhibitors targeting these neddylation enzymes should be discovered to provide more effective treatment for human HCC.

Interestingly, deneddylation enzymes (COPS5, UCHL1, and USP21) were also upregulated, although the global‐protein neddylation was overactivated in HCC. It may be due to the following 2 reasons: (1) The upregulation of deneddylation enzymes may be due to a compensatory effect, and the deneddylation effect of the upregulation of these deneddylation enzymes cannot offset the neddylation effect of the upregulation of the neddylation enzymes in human HCC; and (2) these deneddylation enzymes are not key enzymes in the neddylation pathway in human HCC, and they may affect HCC progression mainly through other pathways. In fact, it has been reported that COPS5 (also known as JAB1) was upregulated in HCC, and loss of COPS5 could inhibit the growth of HCC cells by restoring the expression of p57.^33^ ^,34^ Jun Yu et al found that UCHL1 was expressed in all normal tissues and immortalized normal epithelial cell lines but was low or silenced in 77% (10/13) of HCC cell lines.^35^ UCHL1 suppressed the growth of Huh1 and SNU387 cells and appeared to be a functional tumor suppressor in HCC.^35^ However, they did not detect the expression of UCHL1 in paired human HCC and ANL tissues. They did functional experiments only in 2 HCC cell lines in vitro, without performing studies in vivo. In this study, by qRT‐PCR, Western blotting analyses, and IHC, we found that UCHL1 was significantly upregulated in human HCC tissues, compared with paired ANL tissues and that the upregulation of UCHL1 in HCC was associated with aggressive clinicopathological characteristics and poorer prognosis. Gene Expression Omnibus and The Human Protein Atlas databases also confirmed our results. These findings suggest that UCHL1 may be an oncogene in HCC, which is contradictory to the previous study.^35^ Therefore, the role of UCHL1 in human HCC needs to be further explored. In addition, the role of USP21 in human HCC also requires further studies.

In summary, our research demonstrates that the entire neddylation pathway is overactivated in human HCC and is associated with the survival of patients with HCC. The roles of neddylation and deneddylation enzymes in HCC await further exploration. Moreover, inhibitors targeting neddylation enzymes, except NAE1, should be discovered to provide more effective treatments for human HCC.

## CONFLICT OF INTERESTS

The authors declare no competing financial interests.
