# Supplementary figures and images for: Overactivated neddylation pathway in human hepatocellular carcinoma
Source: Cancer Med. 2018 May 30;7(7):3363–72. doi: 10.1002/cam4.1578 (PMC6051160; doi:10.1002/cam4.1578)

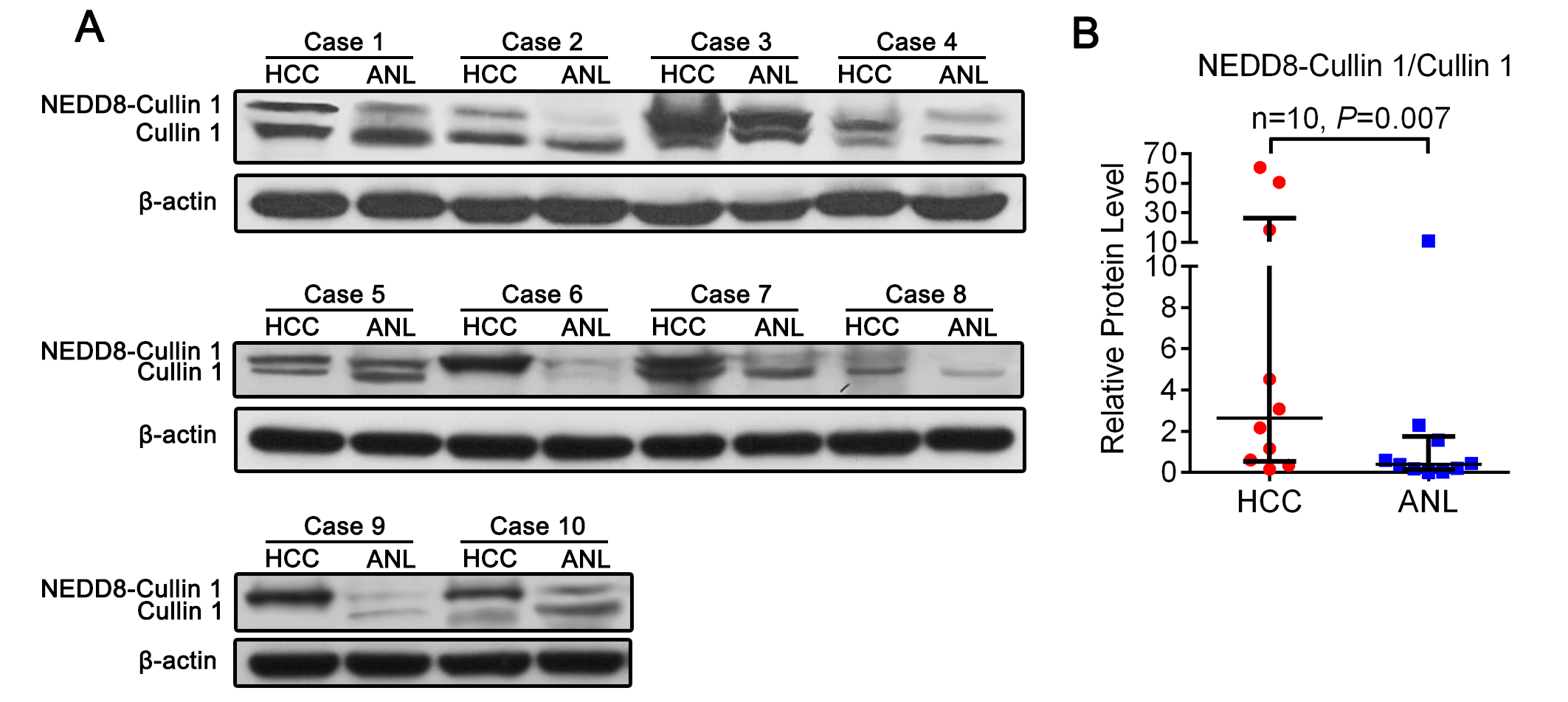

Supplement: Supplementary file 1 [file CAM4-7-3363-s001.tif]

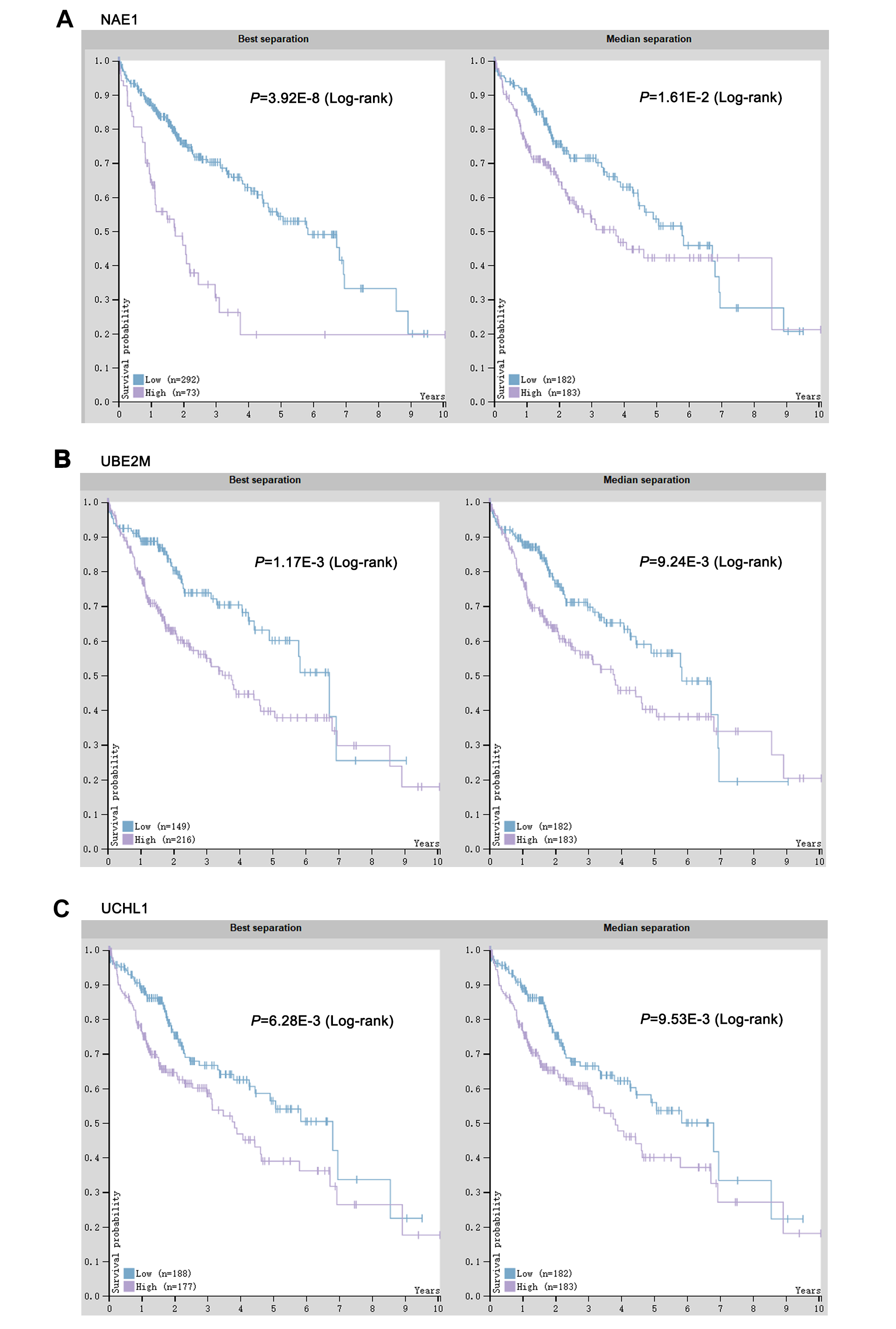

Supplement: Supplementary file 3 [file CAM4-7-3363-s003.tif]
