# Supplementary material for: Overactivated neddylation pathway in human hepatocellular carcinoma
Source: Cancer Med. 2018 May 30;7(7):3363–72. doi: 10.1002/cam4.1578 (PMC6051160; doi:10.1002/cam4.1578)
Supplement: Supplementary file 4 [file CAM4-7-3363-s004.docx]

**Supplementary Table S1. Clinical Characteristics of the HCC Patients in This Study**

|  | Cohort 1 | Cohort 2 | Cohort 3 |
| --- | --- | --- | --- |
| All cases | 306 | 40 | 163 |
| Age, years, ≥60: <60 | 67:239 | 8:32 | 37:126 |
| Gender, male/female | 269:37 | 35:5 | 136:27 |
| HBsAg, positive/negative | 266:40 | 33:7 | 128:35 |
| HBeAg, positive/negative | 64:242 | 10:30 | 42:121 |
| AFP, µg/L, >20: ≤20 | 194:112 | 25:15 | 109:54 |
| Liver cirrhosis, with/without | 249:57 | 8:32 | 115:48 |
| No. tumor, multiple: solitary | 62:244 | 2:38 | 31:132 |
| Tumor size, cm,>5: ≤5 | 150:156 | 25:15 | 88:75 |
| Edmondson’s grade, III+IV: I+II | 275:31 | 37:3 | 131:32 |
| Pathological satellite, present/absent | 227:79 | 18:22 | 66:97 |
| Microvascular invasion, present: absent | 186:120 | 10:30 | 58:105 |
| TNM stage, II+III: I | 66:240 | 13:27 | 101:62 |
| BCLC stage, B+C: 0+A | 62:244 | 1:39 | 116:47 |

Abbreviations: HCC, hepatocellular carcinoma; HBsAg, hepatitis B surface antigen; HBeAg, hepatitis B e antigen; AFP, alpha-fetoprotein; TNM, tumor-node-metastasis; BCLC, Barcelona Clinic Liver Cancer.
