# Supplementary material for: Overactivated neddylation pathway in human hepatocellular carcinoma
Source: Cancer Med. 2018 May 30;7(7):3363–72. doi: 10.1002/cam4.1578 (PMC6051160; doi:10.1002/cam4.1578)
Supplement: Supplementary file 5 [file CAM4-7-3363-s005.docx]

**Supplementary Table S2. The Sequences of PCR Primers Used in This Study**

| **Primer Name** | **Sequence (5'-3')** |
| --- | --- |
| β-actin-F | GGGAAATCGTGCGTGACATTAAG |
| β-actin-R | TGTGTTGGCGTACAGGTCTTTG |
| NAE1-F | GCTCAAGGAGCAGAAGTACGAC |
| NAE1-R | ATGAGCAGATTCTAAAGCCTCTTG |
| UBA3-F | TGGTGTTGGTGCTTGTAA |
| UBA3-R | GTTGTATGCTTCCTCCTCTAA |
| UBE2F-F | CCCCAGATGAGGGTTACTACC |
| UBE2F-R | AGTTTAATCCCCAAACGACATCC |
| UBE2M-F | TGCGGATCCAGAAGGACATA |
| UBE2M-R | GGTCGTCTGGATCTGAGAAGC |
| CBL-F | TCTACATGAAGTGCATCCCATCA |
| CBL-R | AAGAGGACCAGGGCTGAAAGA |
| DCUN1D1-F | TGCCTACTGGAACTTAGTGCT |
| DCUN1D1-R | CTGCAATCATCGTACTGAAGTCT |
| DCUN1D2-F | ACGGCAGGTACAAAGATCCAC |
| DCUN1D2-R | CCACGCTATGACCAATACACTGA |
| DCUN1D3-F | TTGACCCCACAGAATTTCGAG |
| DCUN1D3-R | CAATGCTGTCTGCACTTATTGC |
| FBXO11-F | GTAAACGCTTCAGTGAACTTGC |
| FBXO11-R | GCACCTTTATACAACTGCTGGAA |
| MDM2-F | GAATCATCGGACTCAGGTACATC |
| MDM2-R | TCTGTCTCACTAATTGCTCTCCT |
| RBX1-F | TTGTGGTTGATAACTGTGCCAT |
| RBX1-R | GACGCCTGGTTAGCTTGACAT |
| RNF7-F | TGGAAGACGGAGAGGAAACCT |
| RNF7-R | TCCCCAGACCACAACACAGT |
| ATXN3-F | TATCGCACGTTTTTACAGCAGC |
| ATXN3-R | TCTGGACCCGTCAAGAGAGAA |
| COPS5-F | AACAAGAACAATATCCGCAGGG |
| COPS5-R | CAAGCAATTTGCGATCCAAAGAG |
| SENP8-F | ACTGCGGCAATCAGATGTCTC |
| SENP8-R | GGAACATGGCAATCTCTGCTG |
| UCHL1-F | GCCAATGTCGGGTAGATGA |
| UCHL1-R | GCAAAGTCCCTCCCACAGA |
| UCHL3-F | AGAACGAGCCAGATACCTGGA |
| UCHL3-R | GCTTCCGCCCATCTAATTCAT |
| USP21-F | TTCAGCATACGGACAGAGCC |
| USP21-R | ACGTGTTTCCCAGGTTTCG |
