# Supplementary material for: Overactivated neddylation pathway in human hepatocellular carcinoma
Source: Cancer Med. 2018 May 30;7(7):3363–72. doi: 10.1002/cam4.1578 (PMC6051160; doi:10.1002/cam4.1578)
Supplement: Supplementary file 6 [file CAM4-7-3363-s006.docx]

**Supplementary Table S3. The Antibodies Used in This Study**

| **Antibody** | **Supplier** | **Catalogue number** | **Primary/secondary antibody** | **Host** | **Mono-/polyclonal** |
| --- | --- | --- | --- | --- | --- |
| NEDD8 | CST | #2754 | primary | rabbit | mono |
| NAE1 | Abcam | ab71526 | primary | rabbit | poly |
| UBE2M | Abcam | ab109507 | primary | rabbit | mono |
| Cullin 1 | Abcam | ab75817 | primary | rabbit | mono |
| UCHL1 | Abgent | AM1959B | primary | mouse | mono |
| beta-actin | Sigma-Aldrich | A5441 | primary | mouse | mono |
| IRDye Goat anti-Mouse 680RD | LI-COR | 926-68070 | secondary | goat | poly |
| IRDye Goat anti-Rabbit 800CW | LI-COR | 926-32211 | secondary | goat | poly |
