# Supplementary material for: Overactivated neddylation pathway in human hepatocellular carcinoma
Source: Cancer Med. 2018 May 30;7(7):3363–72. doi: 10.1002/cam4.1578 (PMC6051160; doi:10.1002/cam4.1578)
Supplement: Supplementary file 7 [file CAM4-7-3363-s007.docx]

**Supplementary Table S4. Univariate Analysis of Several Variables for OS and RFS in 306 HCC Patients (Cohort 1)**

| Variable | Overall Survival | |  | Recurrence-Free Survival | |
| --- | --- | --- | --- | --- | --- |
|  | Hazard ratio (95 % CI) | *P*-value |  | Hazard ratio (95 % CI) | *P*-value |
| NEDD8, high | 1.729 (1.228-2.434) | 0.002* |  | 1.890 (1.403-2.544) | <0.001* |
| Age, years, ≥60 | 0.846 (0.558-1.282) | 0.430 |  | 0.962 (0.679-1.362) | 0.826 |
| Gender, male | 0.748 (0.460-1.214) | 0.239 |  | 0.926 (0.593-1.447) | 0.737 |
| HBsAg, positive | 2.200 (1.155-4.189) | 0.016* |  | 2.163 (1.275-3.672) | 0.004* |
| HBeAg, positive | 1.505 (1.032-2.197) | 0.034* |  | 1.631 (1.172-2.270) | 0.004* |
| AFP, >20µg/L | 1.093 (0.772-1.548) | 0.616 |  | 1.253 (0.923-1.702) | 0.147 |
| Liver cirrhosis, with | 0.973 (0.630-1.501) | 0.900 |  | 1.112 (0.755-1.637) | 0.591 |
| No. tumor, multiple | 1.648 (1.126-2.411) | 0.010* |  | 1.484 (1.054-2.091) | 0.024* |
| Tumor size, >5cm | 1.874 (1.332-2.636) | <0.001* |  | 1.545 (1.153-2.070) | 0.004* |
| Edmondson’s grade, III+IV | 1.657 (0.870-3.155) | 0.124 |  | 1.841 (1.047-3.239) | 0.034* |
| Pathological satellite, present | 1.008 (0.689-1.476) | 0.966 |  | 1.189 (0.845-1.674) | 0.321 |
| Microvascular invasion, present | 1.670 (1.163-2.397) | 0.005* |  | 1.519 (1.118-2.066) | 0.008* |
| TNM stage, II+III | 1.746 (1.204-2.532) | 0.003* |  | 1.531 (1.095-2.141) | 0.013* |
| BCLC stage, B+C | 2.111 (1.460-3.053) | <0.001* |  | 2.046 (1.468-2.850) | <0.001* |

Abbreviations: HCC, hepatocellular carcinoma; HBsAg, hepatitis B surface antigen; HBeAg, hepatitis B e antigen; AFP, alpha-fetoprotein; TNM, tumor-node-metastasis; BCLC, Barcelona Clinic Liver Cancer.

* *P*< 0.05
