# Supplementary material for: Overactivated neddylation pathway in human hepatocellular carcinoma
Source: Cancer Med. 2018 May 30;7(7):3363–72. doi: 10.1002/cam4.1578 (PMC6051160; doi:10.1002/cam4.1578)
Supplement: Supplementary file 8 [file CAM4-7-3363-s008.docx]

**Supplementary Table S5. Collinearity Analysis of All the Eight Variables Associated with OS in 306 HCC Patients (Cohort 1)**

| Model | | Unstandardized Coefficients | | Standardized Coefficients | t | Significant | Collinearity Statistics | |
| --- | --- | --- | --- | --- | --- | --- | --- | --- |
|  |  | B | Standard Error | Beta |  |  | Tolerance | Variance Inflation |
| 1 | (Constant) | 0.012 | 0.087 |  | 0.135 | 0.893 |  |  |
|  | NEDD8 | 0.177 | 0.064 | 0.178 | 2.759 | 0.006 | 0.700 | 1.430 |
|  | HBsAg | 0.214 | 0.081 | 0.145 | 2.652 | 0.008 | 0.967 | 1.034 |
|  | HBeAg | 0.187 | 0.067 | 0.153 | 2.792 | 0.006 | 0.964 | 1.037 |
|  | Tumor number | -0.256 | 0.262 | -0.207 | -0.975 | 0.330 | 0.065 | 15.483 |
|  | Tumor size | 0.153 | 0.060 | 0.154 | 2.562 | 0.011 | 0.804 | 1.244 |
|  | Microvascular invasion | 0.007 | 0.066 | 0.007 | 0.103 | 0.918 | 0.696 | 1.436 |
|  | TNM stage | 0.294 | 0.243 | 0.243 | 1.211 | 0.227 | 0.072 | 13.923 |
|  | BCLC stage | 0.154 | 0.102 | 0.124 | 1.504 | 0.134 | 0.424 | 2.358 |

Abbreviations: OS, overall survival; HCC, hepatocellular carcinoma; HBsAg, hepatitis B surface antigen; HBeAg, hepatitis B e antigen; TNM, tumor-node-metastasis; BCLC, Barcelona Clinic Liver Cancer.
