# Supplementary material for: Overactivated neddylation pathway in human hepatocellular carcinoma
Source: Cancer Med. 2018 May 30;7(7):3363–72. doi: 10.1002/cam4.1578 (PMC6051160; doi:10.1002/cam4.1578)
Supplement: Supplementary file 9 [file CAM4-7-3363-s009.docx]

**Supplementary Table S6. Collinearity Analysis of All the Nine Variables Associated with RFS in 306 HCC Patients (Cohort 1)**

| Model | | Unstandardized Coefficients | | Standardized Coefficients | t | Significant | Collinearity Statistics | |
| --- | --- | --- | --- | --- | --- | --- | --- | --- |
|  |  | B | Standard Error | Beta |  |  | Tolerance | Variance Inflation |
| 1 | (Constant) | 0.076 | 0.112 |  | 0.677 | 0.499 |  |  |
|  | NEDD8 | 0.264 | 0.063 | 0.269 | 4.215 | 0.000 | 0.696 | 1.437 |
|  | HBsAg | 0.226 | 0.079 | 0.155 | 2.858 | 0.005 | 0.961 | 1.041 |
|  | HBeAg | 0.227 | 0.065 | 0.188 | 3.475 | 0.001 | 0.964 | 1.038 |
|  | Tumor number | -0.188 | 0.256 | -0.154 | -0.736 | 0.463 | 0.065 | 15.486 |
|  | Tumor size | 0.106 | 0.059 | 0.108 | 1.814 | 0.071 | 0.793 | 1.261 |
|  | Edmondson’s grade | 0.106 | 0.092 | 0.065 | 1.149 | 0.251 | 0.880 | 1.136 |
|  | Microvascular invasion | -0.061 | 0.066 | -0.061 | -0.935 | 0.351 | 0.666 | 1.502 |
|  | TNM stage | 0.146 | 0.237 | 0.123 | 0.618 | 0.537 | 0.072 | 13.925 |
|  | BCLC stage | 0.196 | 0.100 | 0.160 | 1.960 | 0.051 | 0.424 | 2.360 |

Abbreviations: RFS, recurrence free survival; HCC, hepatocellular carcinoma; HBsAg, hepatitis B surface antigen; HBeAg, hepatitis B e antigen; TNM, tumor-node-metastasis; BCLC, Barcelona Clinic Liver Cancer.
