# Supplementary material for: Overactivated neddylation pathway in human hepatocellular carcinoma
Source: Cancer Med. 2018 May 30;7(7):3363–72. doi: 10.1002/cam4.1578 (PMC6051160; doi:10.1002/cam4.1578)
Supplement: Supplementary file 10 [file CAM4-7-3363-s010.docx]

**Supplementary Table S7. Collinearity Analysis of the Seven^#^ Variables Associated with OS in 306 HCC Patients (Cohort 1)**

| Model | | Unstandardized Coefficients | | Standardized Coefficients | t | Significant | Collinearity Statistics | |
| --- | --- | --- | --- | --- | --- | --- | --- | --- |
|  |  | B | Standard Error | Beta |  |  | Tolerance | Variance Inflation |
| 1 | (Constant) | 0.020 | 0.086 |  | 0.228 | 0.820 |  |  |
|  | NEDD8 | 0.185 | 0.064 | 0.186 | 2.910 | 0.004 | 0.708 | 1.412 |
|  | HBsAg | 0.201 | 0.080 | 0.136 | 2.507 | 0.013 | 0.986 | 1.014 |
|  | HBeAg | 0.186 | 0.067 | 0.152 | 2.766 | 0.006 | 0.965 | 1.037 |
|  | Tumor number | 0.039 | 0.098 | 0.031 | 0.395 | 0.693 | 0.460 | 2.172 |
|  | Tumor size | 0.163 | 0.059 | 0.164 | 2.753 | 0.006 | 0.819 | 1.221 |
|  | Microvascular invasion | 0.008 | 0.066 | 0.007 | 0.115 | 0.908 | 0.696 | 1.436 |
|  | BCLC stage | 0.145 | 0.102 | 0.117 | 1.418 | 0.157 | 0.426 | 2.345 |

Abbreviations: OS, overall survival; HCC, hepatocellular carcinoma; HBsAg, hepatitis B surface antigen; HBeAg, hepatitis B e antigen; TNM, tumor-node-metastasis; BCLC, Barcelona Clinic Liver Cancer. #. Excluding TNM stage.
