# Supplementary material for: Overactivated neddylation pathway in human hepatocellular carcinoma
Source: Cancer Med. 2018 May 30;7(7):3363–72. doi: 10.1002/cam4.1578 (PMC6051160; doi:10.1002/cam4.1578)
Supplement: Supplementary file 11 [file CAM4-7-3363-s011.docx]

**Supplementary Table S8. Collinearity Analysis of the Eight^#^ Variables Associated with RFS in 306 HCC Patients (Cohort 1)**

| Model | | Unstandardized Coefficients | | Standardized Coefficients | t | Significant | Collinearity Statistics | |
| --- | --- | --- | --- | --- | --- | --- | --- | --- |
|  |  | B | Standard Error | Beta |  |  | Tolerance | Variance Inflation |
| 1 | (Constant) | 0.081 | 0.112 |  | 0.721 | 0.472 |  |  |
|  | NEDD8 | 0.268 | 0.062 | 0.273 | 4.317 | 0.000 | 0.705 | 1.418 |
|  | HBsAg | 0.219 | 0.078 | 0.150 | 2.802 | 0.005 | 0.979 | 1.021 |
|  | HBeAg | 0.226 | 0.065 | 0.188 | 3.467 | 0.001 | 0.964 | 1.037 |
|  | Tumor number | -0.041 | 0.096 | -0.034 | -0.434 | 0.665 | 0.460 | 2.172 |
|  | Tumor size | 0.111 | 0.058 | 0.113 | 1.920 | 0.056 | 0.809 | 1.236 |
|  | Edmondson’s grade | 0.105 | 0.092 | 0.065 | 1.142 | 0.254 | 0.880 | 1.136 |
|  | Microvascular invasion | -0.061 | 0.065 | -0.060 | -0.928 | 0.354 | 0.666 | 1.502 |
|  | BCLC stage | 0.191 | 0.099 | 0.156 | 1.922 | 0.056 | 0.426 | 2.348 |

Abbreviations: RFS, recurrence free survival; HCC, hepatocellular carcinoma; HBsAg, hepatitis B surface antigen; HBeAg, hepatitis B e antigen; TNM, tumor-node-metastasis; BCLC, Barcelona Clinic Liver Cancer. #. Excluding TNM stage.
