# Supplementary material for: Overactivated neddylation pathway in human hepatocellular carcinoma
Source: Cancer Med. 2018 May 30;7(7):3363–72. doi: 10.1002/cam4.1578 (PMC6051160; doi:10.1002/cam4.1578)
Supplement: Supplementary file 12 [file CAM4-7-3363-s012.docx]

**Supplementary Table S9. Multivariate Analysis of Several Variables for OS and RFS in 306 HCC Patients (Cohort 1)**

| Variable | Overall Survival | |  | Recurrence-Free Survival | |
| --- | --- | --- | --- | --- | --- |
|  | Hazard ratio (95 % CI) | *P*-value |  | Hazard ratio (95 % CI) | *P*-value |
| NEDD8, high | 1.857 (1.314-2.624) | <0.001 |  | 2.050 (1.515-2.774) | <0.001 |
| HBsAg, positive | 2.155 (1.127-4.120) | 0.020 |  | 2.149 (1.261-3.663) | 0.005 |
| HBeAg, positive | 1.507 (1.023-2.220) | 0.038 |  | 1.698 (1.208-2.387) | 0.002 |
| No. tumor, multiple | ━ | NS |  | ━ | NS |
| Tumor size, >5cm | 1.850 (1.298-2.637) | 0.001 |  | 1.581 (1.164-2.147) | 0.003 |
| Edmondson’s grade, III+IV | ━ | ━ |  | ━ | NS |
| Microvascular invasion, present | ━ | NS |  | ━ | NS |
| BCLC stage, B+C | 1.758 (1.194-2.589) | 0.004 |  | 1.745 (1.230-2.474) | 0.002 |

Abbreviations: OS, overall survival; RFS, recurrence free survival; HCC, hepatocellular carcinoma; HBsAg, hepatitis B surface antigen; HBeAg, hepatitis B e antigen; BCLC, Barcelona Clinic Liver Cancer.

NS: no significant
