# Supplementary material for: Overactivated neddylation pathway in human hepatocellular carcinoma
Source: Cancer Med. 2018 May 30;7(7):3363–72. doi: 10.1002/cam4.1578 (PMC6051160; doi:10.1002/cam4.1578)
Supplement: Supplementary file 13 [file CAM4-7-3363-s013.docx]

**Supplementary Table S10. The Expression of Neddylation and De-neddylation Enzymes**

**in 214 Paired HCC and ANL Tissues According to GSE14520-GPL3921 Using GEO2R**

| Gene.symbol | ID | GB_ACC | Adj. P.Val | P.Value | Log FC | Fold change (HCC/ANL) | Gene.title |
| --- | --- | --- | --- | --- | --- | --- | --- |
| ATXN3 | 205416_s_at | NM_004993 | 5.19E-08 | 2.18E-08 | 0.168 | 1.123 | ataxin 3 |
| CBL | 206607_at | NM_005188 | 8.18E-01 | 7.98E-01 | 0.004 | 1.003 | Cbl proto-oncogene |
| COPS5 | 201652_at | NM_006837 | 2.37E-40 | 1.72E-41 | 0.853 | 1.806 | COP9 signalosome subunit 5 |
| DCUN1D1 | 218583_s_at | NM_020640 | 1.19E-01 | 9.86E-02 | 0.088 | 1.063 | defective in cullin neddylation 1 domain containing 1 |
| DCUN1D2 | N/A | N/A | N/A | N/A | N/A | N/A | N/A |
| DCUN1D3 | N/A | N/A | N/A | N/A | N/A | N/A | N/A |
| FBXO11 | 219208_at | NM_025133 | 1.59E-13 | 4.51E-14 | 0.192 | 1.142 | F-box protein 11 |
| MDM2 | 205386_s_at | NM_002392 | 6.46E-01 | 6.13E-01 | -0.027 | 0.982 | MDM2 proto-oncogene |
| NAE1 | 202268_s_at | NM_003905 | 2.18E-41 | 1.51E-42 | 0.816 | 1.760 | NEDD8 activating enzyme E1 subunit 1 |
| RBX1 | 218117_at | NM_014248 | 3.38E-53 | 1.18E-54 | 0.723 | 1.651 | ring-box 1 |
| RNF7 | 218286_s_at | NM_014245 | 7.64E-30 | 9.16E-31 | 0.567 | 1.481 | ring finger protein 7 |
| SENP8 | N/A | N/A | N/A | N/A | N/A | N/A | N/A |
| UBA3 | 209115_at | AL117566 | 4.47E-22 | 7.87E-23 | 0.496 | 1.410 | ubiquitin like modifier activating enzyme 3 |
| UBE2F | N/A | N/A | N/A | N/A | N/A | N/A | N/A |
| UBE2M | 203109_at | NM_003969 | 3.45E-24 | 5.46E-25 | 0.642 | 1.561 | ubiquitin conjugating enzyme E2 M |
| UCHL1 | 201387_s_at | NM_004181 | 3.15E-07 | 1.41E-07 | 0.594 | 1.509 | ubiquitin C-terminal hydrolase L1 |
| UCHL3 | 204616_at | NM_006002 | 1.42E-03 | 9.09E-04 | 0.229 | 1.172 | ubiquitin C-terminal hydrolase L3 |
| USP21 | 218367_x_at | NM_012475 | 1.25E-29 | 1.51E-30 | 0.595 | 1.511 | ubiquitin specific peptidase 21 |

**N/A: The expression of this gene can not be obtained from this microarray (GPL3921).**
